# Validation of the Fitbit Charge 2 compared to the ActiGraph GT3X+ in older adults with knee osteoarthritis in free-living conditions

**DOI:** 10.1371/journal.pone.0211231

**Published:** 2019-01-30

**Authors:** Jamie E. Collins, Heidi Y. Yang, Taylor P. Trentadue, Yusi Gong, Elena Losina

**Affiliations:** 1 Orthopaedic and Arthritis Center for Outcomes Research and Policy and Innovation eValuation in Orthopaedic Treatments (PIVOT) Center, Department of Orthopaedic Surgery, Brigham and Women’s Hospital, Boston, Massachusetts, United States of America; 2 Harvard Medical School, Boston, Massachusetts, United States of America; 3 Division of Rheumatology, Immunology and Allergy, Brigham and Women’s Hospital, Boston, Massachusetts, United States of America; 4 Department of Biostatistics, Boston University School of Public Health, Boston, Massachusetts, United States of America; UNSW Sydney, AUSTRALIA

## Abstract

**Objective:**

To evaluate physical activity (PA) and sedentary time in subjects with knee osteoarthritis (OA) measured by the Fitbit Charge 2 (Fitbit) and a wrist-worn ActiGraph GT3X+ (AGW) compared to the hip-worn ActiGraph (AGH).

**Design:**

We recruited a cohort of subjects with knee OA from rheumatology clinics. Subjects wore the AGH for four weeks, AGW for two weeks, and Fitbit for two weeks over a four-week study period. We collected accelerometer counts (ActiGraphs) and steps (ActiGraphs, Fitbit) and calculated time spent in sedentary, light, and moderate-to-vigorous activity. We used triaxial PA intensity count cut-points from the literature for ActiGraph and a stride length-based cadence algorithm to categorize Fitbit PA. We compared Fitbit wear times calculated from a step-based algorithm and a novel algorithm that incorporates steps and heart rate (HR).

**Results:**

We enrolled 15 subjects (67% female, mean age 68 years). Relative to AGH, Fitbit, on average, overestimated steps by 39% and sedentary time by 37% and underestimated MVPA by 5 minutes. Relative to AGH, AGW overestimated steps 116%, underestimated sedentary time by 66%, and captured 281 additional MVPA minutes. The step-based wear time Fitbit algorithm captured 14% less wear time than the HR-based algorithm.

**Conclusions:**

Fitbit overestimates steps and underestimates MVPA in knee OA subjects. Cut-offs validated for AGW should be developed to support the use of AGW for PA assessment. The HR-based Fitbit algorithm captured more wear time than the step-based algorithm. These data provide critical insight for researchers planning to use commercially-available accelerometers in pragmatic studies.

## Introduction

Physical activity (PA) is associated with lower all-cause mortality and improved quality of life[1–4]. Guidelines outlined by the Centers for Disease Control and Prevention (CDC) recommend that older adults engage in ≥150 minutes of moderate-intensity PA, 75 minutes of vigorous-intensity PA, or a combination of the two weekly[5]. Knee osteoarthritis (OA), affecting over 14 million Americans,[6] is a leading cause of disability among older adults[7]. PA may help alleviate pain and improve function in knee OA[8]. However, <13% of men and <8% of women with symptomatic knee OA meet CDC PA guidelines[9].

Accelerometers capture objectively-measured PA in free-living conditions[10]. ActiGraphs—including the 7164, GT1M, and GT3X models—have been used extensively in OA research[9, 11–15]. Thresholds for light, moderate, and vigorous PA have been established for hip-worn ActiGraphs, but the device can be used on wrist and ankle in addition to hip. Consumer-marketed wearable activity monitors (“wearables”), such as the Fitbit, have recently gained popularity[16, 17]. The monitors are straightforward to wear, provide real-time activity metrics to users, and can transmit data remotely via Bluetooth syncing. The availability of relatively inexpensive, commercially-available wearables that capture motion and heart rate (HR) offers an opportunity to capture PA over long time horizons.

Given the high prevalence of sedentary behaviors, especially among those with knee OA,[14, 18] there is a growing need for PA interventions that include scalable means to measure activity. Given differences in gait mechanics in OA patients,[19] it is important to understand how to measure PA in free-living conditions in this patient population. While ActiGraphs are considered the gold-standard,[20–22] they are expensive and can be purchased exclusively for medical or scientific use[23]. Further, while ActiGraph data must be downloaded using specific software, Fitbit data can be uploaded from an individual’s computer or mobile device and downloaded remotely. Thresholds for ActiGraph counts determining the light, moderate, and vigorous PA have been defined for hip-worn devices[24–26]. These features make Fitbits appealing for interventions to improve PA. However, using Fitbits to objectively measure PA in interventions must be supported by evidence of validity compared to the ActiGraph.

While several studies in knee OA patients have used Fitbits, none, to our knowledge, has attempted to compare Fitbit estimates to ActiGraph-based PA measures in free-living conditions. Furthermore, most Fitbit wearables are wrist-worn, while recommended ActiGraph placement is on the hip. We designed a validation study to establish the accuracy of a wrist-worn Fitbit Charge 2 compared to the hip-worn ActiGraph in measuring time spent sedentary and in light and moderate-to-vigorous physical activity (MVPA). We further examined differences in Fitbit and ActiGraph assessments derived from accelerometers worn at the same site (wrist) among knee OA patients. Understanding the accuracy of Fitbits compared to ActiGraphs is necessary to design, test, and implement pragmatic PA interventions for persons with knee OA.

## Materials and methods

### Participants

We identified knee OA patients using schedules of four rheumatologists at Brigham and Women’s Hospital, Boston, MA. We screened electronic medical records (EMRs) for initial inclusion criteria: age between 50 and 85 years; primary diagnosis of knee OA; and English as primary language. Exclusion criteria were: diagnoses of inflammatory arthritides or Parkinson’s disease; history of total knee replacement (TKR); ambulation using a wheelchair; or recent surgery precluding participation.

Subjects meeting the EMR criteria screen received a phone call, during which we described the study and performed additional exclusion criteria screening for adverse skin reactions to Velcro, workers’ compensation for a knee-related symptom, inability to walk safely, or no access to a computer, tablet, or smartphone. The study was approved by the Partners Institutional Review Board (IRB). All study participants signed the informed consent forms provided to them. Recruitment took place from March through July 2017. The enrollment schematic is presented in Fig C in S1 File.

### Instruments and measures

#### Fitbit charge 2

Fitbit Charge 2 (Fitbit Inc., San Francisco, CA) is a wireless, wrist-worn, triaxial accelerometer. A proprietary algorithm translates raw acceleration signals into steps and activity levels[27]. It estimates steps, HR, activity level, and energy expenditure each minute.

#### ActiGraph GT3X+

ActiGraph GT3X+ (ActiGraph Corp., Pensacola, FL) is a medical-grade, triaxial accelerometer that provides activity counts and steps. It has been validated to provide objective measures of sedentary behavior and PA in free-living conditions and can be worn on the wrist (AGW) or hip (AGH)[10, 25, 26]. Using proprietary algorithms, ActiGraph’s software computes PA level, energy expenditure, and metabolic equivalents of task (METs). We integrated over one-minute epochs to match Fitbit’s minute-level data output.

### Study procedures

Subjects attended a baseline visit and completed a questionnaire about cardiovascular-related comorbidities, medication use, and the Knee injury and Osteoarthritis Outcome Score (KOOS)[28]. We measured the distance traveled in 30 steps to estimate stride length. Subjects were given two ActiGraphs, one hip-worn (AGH) and one wrist-worn (AGW), and a Fitbit Charge 2 and were instructed how to wear and charge the devices and synchronize the Fitbit via Bluetooth. They were asked to wear devices for ≥10 hours daily while awake[10]. Participants were instructed to wear the Fitbit and AGW on their nondominant wrists and the AGH along the midaxillary line level with the iliac crest. We provided wear instructions verbally and with written materials.

The study period lasted four weeks. In the first week, participants were asked to wear only AGH. AGW was added during the second week to control for differential wear position of the AGH and Fitbit. During the third week, we asked subjects to wear all three accelerometers, which switched to AGH and Fitbit for week four.

During the study, we sent daily reminders to participants via e-mail or text message to wear the appropriate monitor(s). To encourage adherence, participants received between $3 and $7 per day dependent on number of devices worn. We instructed participants to sync the Fitbit every three days during Fitbit wear. We downloaded these minute-level data weekly from each participant's Fitbit using a custom, in-house Python program incorporating the Fitbit application programming interface. At the end of the study, subjects returned devices in pre-paid envelopes. We downloaded ActiGraph data using ActiLife (ActiLife v6.13.3, ActiGraph Corp., Pensacola, Florida). See additional study design in Tables D and E in S1 File.

### Outcomes measured and data processing

We defined a valid day as ≥10 hours of wear and week as ≥4 valid days.[10] The primary outcomes of interest were steps and minutes of sedentary time and MVPA. Both the Fitbit and ActiGraphs measure steps per day. Daily sedentary time, MVPA, and wear time were calculated for each device as described below.

#### Fitbit data processing

In the primary analysis, we calculated Fitbit wear time using both step and HR data (HR-based algorithm). Any minute when either HR or steps were greater than zero was categorized as wear time. In the algorithm that incorporates steps but not HR (step-based algorithm), non-wear time was identified as bouts of ≥60 consecutive minutes with zero steps[20]. We subtracted non-wear time from 24 hours to calculate total daily wear time. We compared the wear time results from both algorithms with one another and with AGH.

Previously, the recommendation of 100 steps per minute in interrupted bouts of ≥10 minutes was the MVPA threshold for healthy, younger adult populations[29, 30]. However, because older adults expend less energy reaching 3 METs than younger adults,[31, 32] we calculated individualized cadence-based (steps per minute) MVPA thresholds. We defined MVPA as expending 3 METs of energy, corresponding to walking at 2.5 kilometers per hour for older adults[31]. We divided this speed by half of stride length, an approximation of step length,[33] to calculate personalized MVPA thresholds. Using a rolling window algorithm, we defined MVPA in bouts of ≥10 minutes, allowing two grace minutes, wherein cadence was greater than the threshold. We calculated sedentary time by summing minutes when step count was zero but HR was non-missing. Light activity was defined as wear time not classified as sedentary time or MVPA.

#### ActiGraph data processing

For AGH and AGW, we calculated minute-level vector magnitude (VM) counts from three ActiGraph axes by taking the square root of the sum of the squares. We calculated ActiGraph wear time using an algorithm to search through minute-level data for counts >0, indicative of wear[10]. We identified consecutive bouts of non-wear ≥60 minutes and subtracted non-wear time from 24 hours to calculate daily wear time[10], We defined ActiGraph-measured MVPA as VM ≥1924 counts per minute in bouts of 10 minutes, allowing for two grace minutes where the VM counts could be <1924[31]. We calculated daily sedentary time by summing minutes with 0<VM<200 counts[34]. Light activity was defined as wear minutes not classified as sedentary activity or MVPA. We used the same threshold for both wrist-and hip-worn ActiGraph as no published thresholds exist for wrist-worn ActiGraph.

#### MVPA guidelines

For each device and person-week, we determined whether subjects met weekly CDC-recommended guidelines of ≥150 MVPA minutes [5] and the intermediate MVPA recommendation of ≥45 minutes as suggested by OA literature[35].

### Participant satisfaction

At the end of the wear period, we sent participants a survey inquiring about ease of wear, instructions, and discomfort.

### Statistical analyses

The primary comparisons were Fitbit versus AGH and AGW versus AGH, as AGH is the gold-standard reference device[10, 25]. As a secondary analysis, we evaluated AGW versus Fitbit, keeping the site of accelerometer wear constant. For day-level outcomes (steps, MVPA, and sedentary time), we deemed valid person-days for each comparison if both devices had valid days and if the difference in wear time between the devices was ≤60 minutes.

We present descriptive statistics and scatterplots for continuous variables. We computed intra-class correlation coefficients (ICC) to compare step counts in our primary comparison of AGH versus Fitbit. We first calculated ICCs for each subject and then a weighted average based on number of days of device wear. For steps and sedentary time, we computed percent bias compared to AGH. We anticipated many person-days to have 0 minutes of MVPA;[9] thus, we did not compute percent bias and instead created a dichotomous indicator of whether any MVPA was recorded that day. For binary variables, we calculated frequencies for all device comparisons.

We conducted analyses for day-level observations for both Fitbit and AGW versus AGH stratified by steps <7,500 and ≥7,500 steps per day as measured by AGH[30]. Statistical analyses were done in SAS 9.4 (SAS Institute Inc., Cary, NC).

## Results

### Cohort characteristics

We identified 80 knee OA patients, 53 of whom passed EMR screen. We were unable to contact 14 subjects, and 22 were uninterested in participating. Two subjects were ineligible at the phone screen: one did not have access to a Bluetooth-enabled device, and one had filed for workers’ compensation. Those who were not enrolled had similar age and sex distributions compared to participants.

Fifteen subjects were eligible and agreed to participate. Sample characteristics are presented in Table 1. The mean (standard deviation [SD]) age was 68 (8) years and ranged from 54 to 80. The mean (SD) BMI was 30 (6) kg/m^2^; 40% had BMI >30 kg/m^2^. The participants were 73% white and 67% female. The most prevalent comorbidities were hypertension (67%) and diabetes (13%). Eighty-seven percent of participants reported taking ≥1 medication for knee pain in the previous week: 60% used NSAIDs, 40% acetaminophen, and 13% opioids. The mean KOOS pain, function, symptom, and quality of life scores, respectively, were 50, 54, 50, and 38 (0–100: worst to best).[28] The mean (SD) stride length was 1.2 (0.2) meters. The average cadence needed to reach moderate PA was 71 steps per minute, ranging from 57 to 136 steps per minute; one subject who used a walker had a MVPA cadence >100 steps per minute.

**Table 1:** Baseline demographic, biometric, and clinical characteristics for sample (n = 15).

Characteristic	Value*
Age (years)	68 (8), 68
Female sex, n (%)	10 (67)
BMI (kg/m^2^)	30 (6), 28
Obese (BMI >30)	6 (40)
Stride length (m)	1.2 (0.2), 1.2
Comorbidities, n (%)	
Hypertension	10 (67)
History of stroke	1 (7)
Diabetes mellitus	2 (13)
Medication for knee pain in past week, n (%)	13 (87)
KOOS scores†	
Pain	50 (20), 58
Function	54 (20), 56
Symptoms	50 (17), 51
Quality of life	38 (17), 40

*Values are mean (SD), median unless otherwise specified.

†KOOS scores: 0–100; 0 represents the worst outcome score.[28]

### Day-level physical activity measurements

Fifteen participants contributed AGH and AGW data; 14 provided Fitbit data. There were 404 person-days of data for AGH, 201 for AGW, and 196 for Fitbit, of which 373 (92%) person-days for AGH, 184 (92%) for AGW, and 160 (82%) for Fitbit were valid. We used the HR-based algorithm to define valid Fitbit days for comparisons. We present linear regression equations for pairwise comparisons in Fig A in S1 File.

#### AGH vs. Fitbit

**Fig 1:**
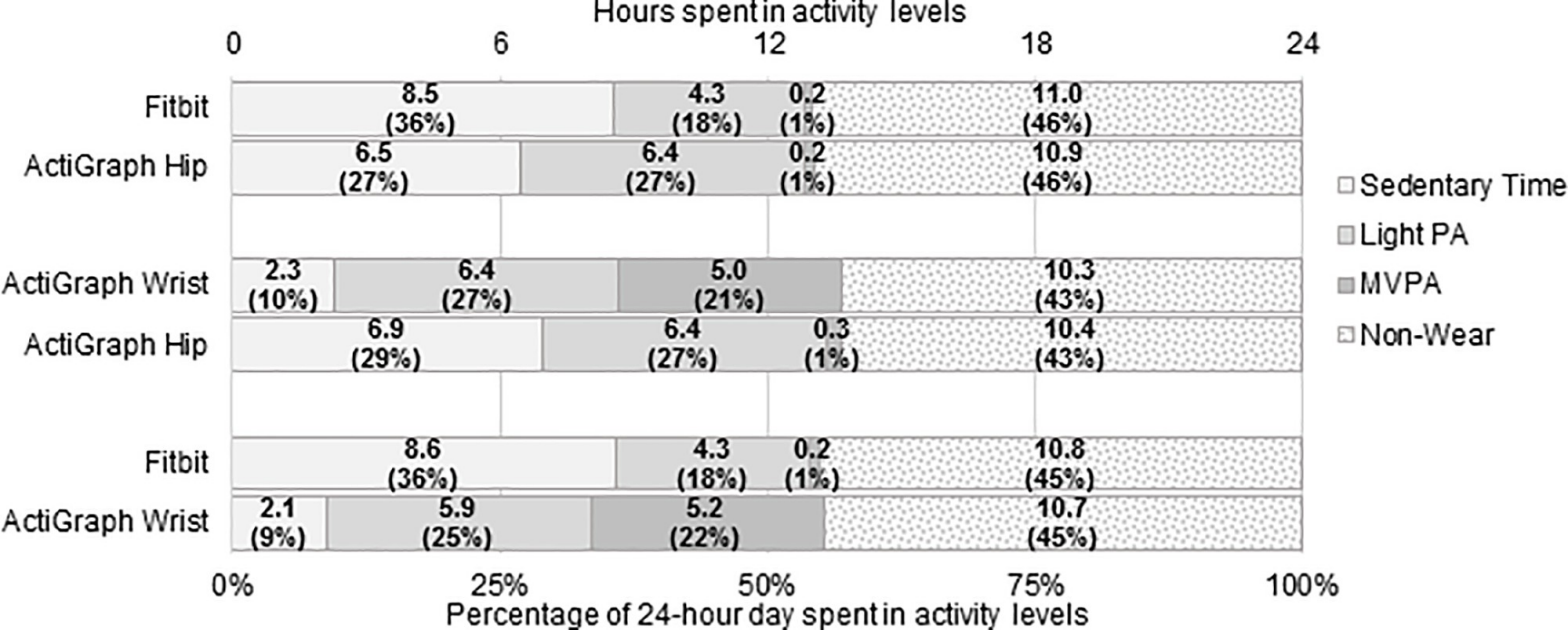
Distribution of time and percentage of the day spent in sedentary time, light activity, moderate-to-vigorous PA (MVPA), and non-wear time per device comparison. Each comparison includes only observations in which the wear time difference between the two devices was ≤60 minutes.

**Fig 2:**
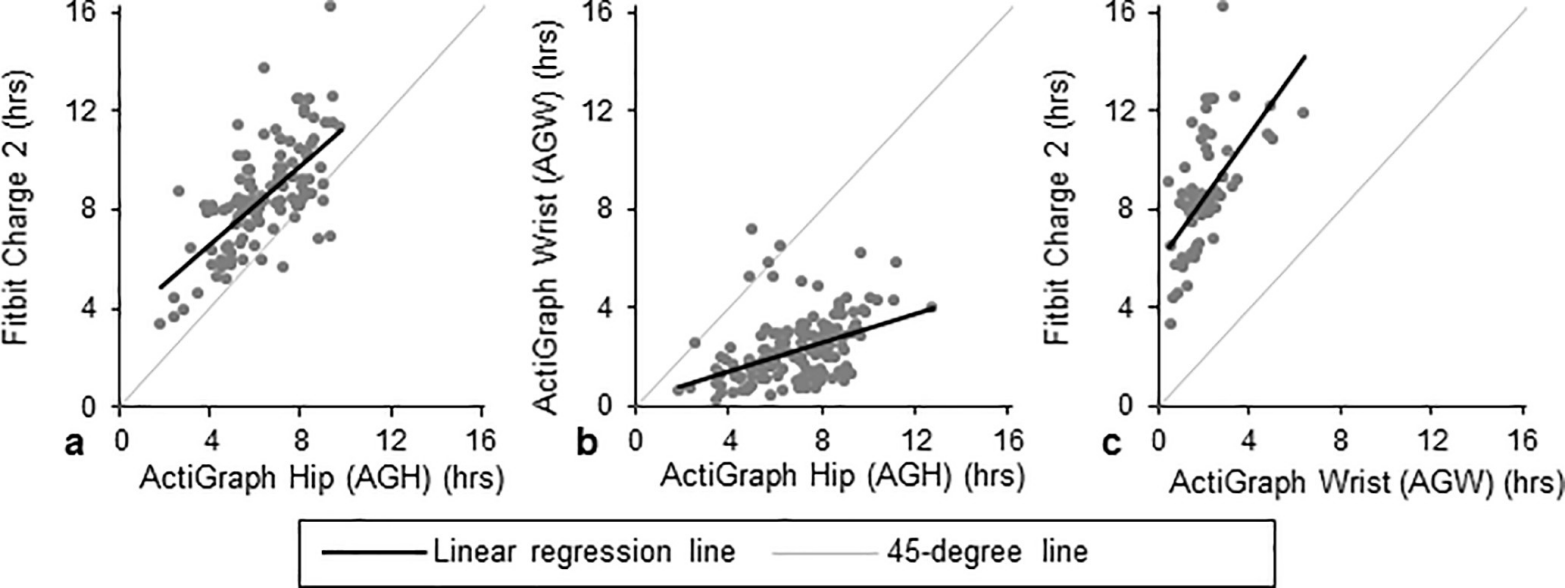
Pairwise comparisons of hours in sedentary behavior per day. **The black line represents the regression line, and the grey line represents a 45-degree line.** Comparisons for Fitbit versus ActiGraph hip (AGH), ActiGraph wrist (AGW) versus AGH, and Fitbit versus AGW are presented in panels a, b, and c, respectively.

**Fig 3:**
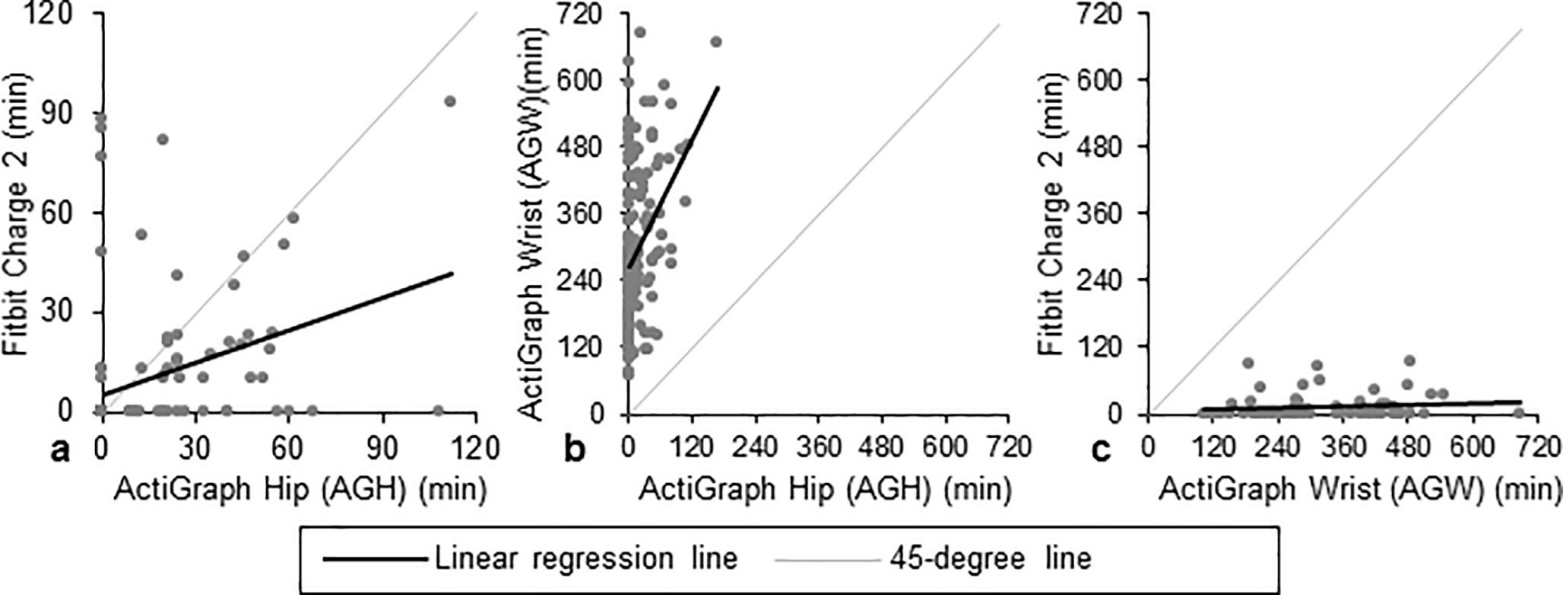
Pairwise comparisons of daily minutes in moderate-to-vigorous physical activity (MVPA). The black line represents the regression line, and the grey line represents a 45-degree line. Comparisons for Fitbit versus ActiGraph hip (AGH), ActiGraph wrist (AGW) versus AGH, and Fitbit versus AGW are presented in panels a, b, and c, respectively.

**Fig 4:**
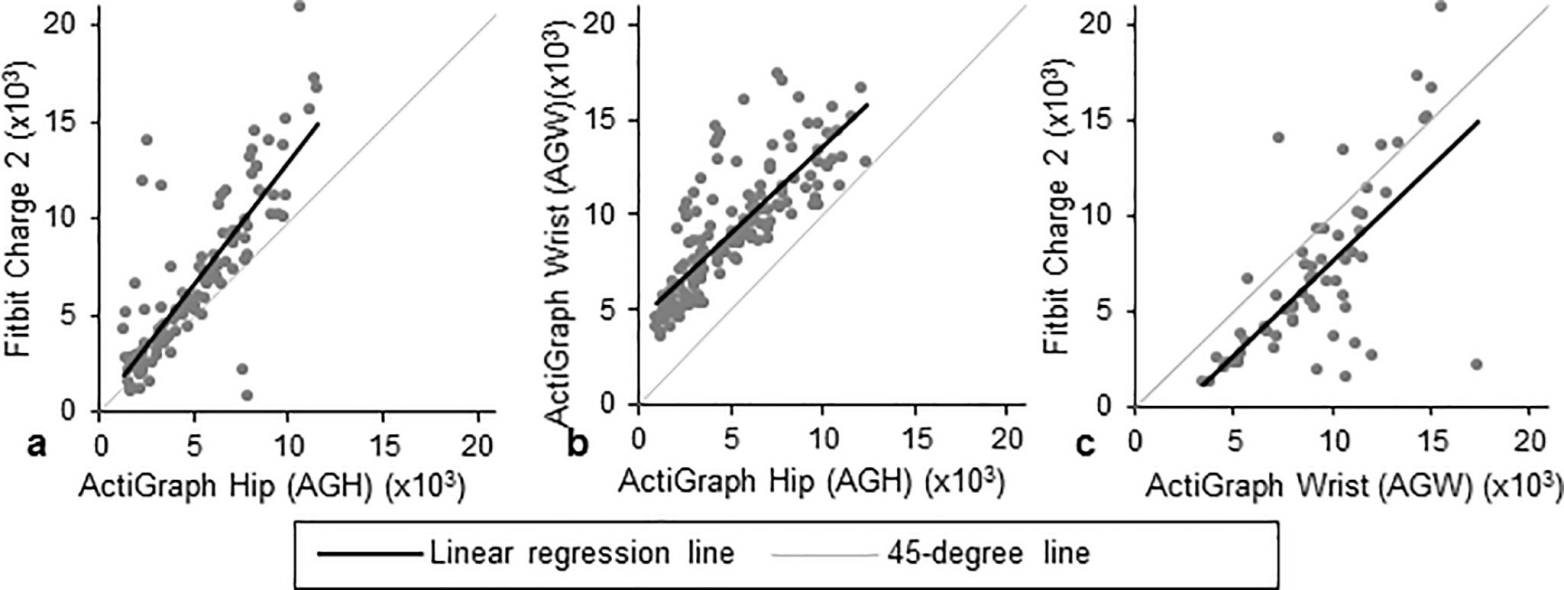
Pairwise comparisons of steps per day as measured by each activity monitor. The black line represents the regression line, and the grey line represents a 45-degree line. Comparisons for Fitbit versus ActiGraph hip (AGH), ActiGraph wrist (AGW) versus AGH, and Fitbit versus AGW are presented in panels a, b, and c, respectively.

There were 28 valid person-weeks in which both devices were worn, resulting in 152 person-days wherein both AGH and Fitbit were worn for ≥10 hours. Of these, 114 (75%) had difference in wear time ≤60 minutes. Per AGH’s measurements, subjects spent mean (SD) 6.5 (1.8) hours per day sedentary, 6.4 (2.0) hours in light activity, and 0.2 (0.4) hours in MVPA (Fig 1). Per Fitbit’s measurements, participants spent 8.5 (2.2) hours sedentary (Fig 2), 4.3 (1.4) hours in light activity (Fig A in S1 File), and 0.2 (0.3) hours in MVPA (Fig 3). Fitbit captured mean (SD) 6,732 (4,155) steps versus 5,084 (2,687) on AGH, with an average difference in steps of 1,648 (2,514) (39% bias; Fig 4). Fitbit recorded, on average, 5 fewer minutes of MVPA than AGH. Median minutes of MVPA for both devices was 0. Both devices recorded zero minutes of MVPA in 50% of all valid comparison days, and at least one device recorded zero MVPA minutes in 76% of all valid comparison days. Fitbit overestimated sedentary time by 37% compared to the AGH, capturing mean (SD) 2.1 (1.7) more hours (Table 2). The ICC for steps was 0.602.

**Table 2:** Comparison of mean day-level physical activity measured by the Fitbit, wrist ActiGraph (AGW), and hip ActiGraph (AGH).

	Fitbit vs. ActiGraph Hip		ActiGraph Wrist vs. ActiGraph Hip	
N (person-days)	114		143	
	Fitbit	AGH	AGW	AGH
Sedentary time (minutes)				
Mean	511	387	138	416
SD	129	106	80	117
Median	500	383	126	431
Difference vs. AGH	124	-	-279	-
% bias vs. AGH	37	-	-66	-
MVPA, bouted (minutes)				
Mean	10	14	299	18
SD	21	22	142	28
Median	0	0	279	0
Difference vs. AGH	-5	-	281	-
Steps				
Mean	6,732	5,084	9,131	5,102
SD	4,155	2,687	3,349	2,966
Median	5,661	4,692	8,913	4,357
Difference vs. AGH	1,648	-	4,029	-
% bias vs. AGH	39	-	116	-
Person-weeks (n [%]) achieving MVPA thresholds	28		28	
≥150 min/week	2 (7)	7 (25)	28 (100)	8 (29)
≥45 min/week	11 (39)	14 (50)	28 (100)	15 (54)

Fitbit recorded 2 (7%) person-weeks where CDC MVPA guidelines were met, while AGH recorded 7 (25%). Changing this threshold to ≥45 minutes per week resulted in 11 (39%) person-weeks meeting the MVPA threshold as recorded by the Fitbit and 14 (50%) by AGH.

#### AGH vs. AGW

There were 28 valid person-weeks resulting in 174 days when both AGW and AGH were worn ≥10 hours. Of these, there were 143 valid comparison days where the difference in wear time was ≤60 minutes. AGW recorded mean 2.3 (1.3) hours sedentary, 6.4 (1.8) hours in light activity, and 5.0 (2.4) hours in MVPA (Fig 1). AGH recorded participants spending mean (SD) 6.9 (1.9) hours per day sedentary (Fig 2), 6.4 (2.1) hours in light activity (Fig A in S1 File), and 0.3 (0.5) hours in MVPA (Fig 3). Mean (SD) steps per day was 9,131 (3,349) on AGW versus 5,102 (2,966) on AGH with average step difference 4,029 (2,036) (116% bias; Fig 4). AGW, on average, recorded 281 additional MVPA minutes versus AGH. AGW captured ≥68 minutes of MVPA in all valid comparison days, while AGH captured zero minutes of MVPA in 68% of all comparison days. AGW captured 66% less sedentary time than AGH, underestimating sedentary time by a mean (SD) of 4.6 (1.8) hours (Table 2).

AGW recorded 28 (100%) person-weeks meeting CDC MVPA guidelines, while AGH recorded 8 (29%) person-weeks in which participants met guidelines. When the threshold was changed to ≥45 weekly MVPA minutes, 28 (100%) met guidelines per AGW compared to 15 (54%) per AGH.

#### AGW vs. Fitbit

There were 75 days when both Fitbit and AGW were worn ≥10 hours; of these, there were 62 (83%) valid comparison days when the difference in wear time was ≤60 minutes. AGW recorded mean (SD) 2.1 (1.1) hours in sedentary activity, 5.9 (1.5) hours in light activity, and 5.2 (2.2) hours in MVPA per day (Fig 1). Fitbit recorded 8.6 (2.4) hours sedentary (Fig 2), 4.3 (1.4) hours in light activity (Fig A in S1 File), and 0.2 (0.4) hours in MVPA (Fig 3). AGW averaged 2,355 (3,274) more steps (-29% bias; Fig 4) and 158 (93) additional minutes of MVPA compared to Fitbit. There were no days when AGW recorded 0 minutes of MVPA while Fitbit recorded >0, and there were 39 (63%) of days when AGW recorded >0 minutes of MVPA while Fitbit recorded 0. Sedentary time was higher with Fitbit than AGW by mean (SD) 6.5 (2.0) hours.

There were 14 valid person-weeks. Of these, AGW recorded 14 (100%) person-weeks in which participants met CDC MVPA guidelines, while the Fitbit recorded 2 (14%) meeting guidelines. When the threshold was lowered to ≥45 minutes of MVPA, AGW recorded 14 (100%) meeting guidelines compared to 6 (43%) by Fitbit.

#### Fitbit: Step-only versus HR wear time algorithm

There were 196 person-days wherein subjects were asked to wear the Fitbit. Using the step-based algorithm, we estimated mean (SD) 9.4 (4.5) daily Fitbit wear hours. When incorporating HR as an indicator of wear status, wear time increased to 11.1 (4.9) hours (Fig B in S1 File). The Fitbit step-based algorithm underestimated wear time compared to the HR-based algorithm (9.4 versus 11.1 hours; 14% bias).

#### Stratified analyses

The results of the stratified analyses are similar to those reported in the combined analysis; the subset of person-days with <7,500 and ≥7,500 steps per day are presented in Tables B and C in S1 File.

### Participant experience

Eleven participants (73%) returned the participant experience questionnaire. All indicated that the wear instructions were clear. Of responding subjects, 100%, 91%, and 82% indicated that Fitbit, AGW, and AGH, respectively, were easy to wear. Two participants (18%) experienced some discomfort wearing the devices.

## Discussion

We compared measures of PA obtained from the Fitbit Charge 2 and wrist-worn ActiGraph GT3X+ against the hip-worn ActiGraph in older adults with knee OA. Compared to AGH, we found that Fitbit overestimates steps by 39% and that AGW overestimates steps by 116%; Fitbit overestimates daily sedentary time by 37% while AGW underestimates sedentary time by 66%; and Fitbit underestimates daily MVPA by 50% while AGW reported considerably more MVPA. Our results confirm previous findings that AGW records significantly more steps than AGH[36] and that Fitbit sometimes overestimates steps in free-living settings[37, 38]. The overestimation of steps and MVPA suggests that in order to use AGW to measure PA, specific thresholds should be established and thresholds derived from hip-worn ActiGraphs should not be used to measure PA by wrist-worn ActiGraphs in older adults with OA. Our findings differ from literature reporting that Fitbit underestimates sedentary time[37]; this may be attributed, in part, to our specific sample of participants with musculoskeletal conditions that limit mobility and impact gait and, in part, to our consideration of HR, which allows us to clearly distinguish no wear from sedentary time.

Studies on younger and middle-aged adults show high correlation and little systematic difference between the step outputs of Fitbit and AGH;[20, 39] in older adults, however, Fitbit overestimates steps compared to AGH[40–42]. Paul et al. reported that Fitbit showed higher agreement with physiotherapist-assessed steps than AGH (interclass coefficient versus physiotherapist: 0.88 for Fitbit; 0.60 for AGH)[42]. This suggests that, while AGH is the gold standard in PA research, its step classification algorithm may not be the most appropriate for capturing steps in older adults with musculoskeletal disease.

Farina et al. [40] compared the Fitbit Charge HR to the hip-worn ActiGraph GT3X+ in a population of older adults in free-living conditions. The authors reported good agreement in, but systematic overestimation of, step counts by Fitbit: the Fitbit recorded, on average, 36% more steps than AGH, similar to the 39% step overestimation in our study[40]. However, work by Dominick et al. [20] found a 7% Fitbit step count overestimation versus AGH. This discrepancy may be due to differences in study population and data processing algorithms. The Dominick et al. cohort included ostensibly healthy younger adults. Because adults with OA have characteristic gait mechanics that includes attenuated peak forces,[19] differences in step counts may be driven by proprietary Fitbit acceleration thresholds used to classify steps[43]. Additionally, Dominick et al. did not restrict analyses to days wherein inter-device wear time agreement was within 60 minutes. As differences in wear time may contribute to discrepancies in daily step and activity counts, we incorporated a measure of wear time similarity in our analysis. We found lower correlation between AGH and Fitbit compared to other studies;[40, 44] this may be due to differences in cohort characteristics, as our cohort was an older, less active patient sample with mobility impairments.

In addition to capturing steps, Fitbit reports measures of PA, such as PA intensity and sedentary time, using a proprietary algorithm. While we did not incorporate these Fitbit-reported measures in our study and instead used a cadence-based approach, Dominick et al.[20] used the Fitbit PA algorithms in a cohort of younger adults and found modest relationships between devices: Fitbit underestimated time spent in both sedentary and light activity but slightly overestimated MVPA compared to AGH. Using our HR- and cadence-based algorithm, we found that Fitbit overestimated sedentary time and light activity but underestimated time in MVPA. Both studies found modest agreement between the devices, but the trends for sedentary time and MVPA differed, potentially due to Fitbit data processing—Fitbit versus our cadence-based algorithm—or variation in cohort characteristics, with our study including older adults with musculoskeletal impairments.

Our study uses novel algorithms for Fitbit processing that incorporate metabolic differences between younger adults, the cohort in whom many PA cut-points were validated, and older adults. The established 100 steps per minute cadence for moderate PA has been established in several laboratory studies[29, 45]. However, 14/15 subjects in our study required fewer than 100 steps per minute to reach 2.5 kilometers per hour; the subject whose threshold exceeded this cadence ambulated with assistance from walker. We captured more minutes of MVPA from Fitbit using individualized cadences than we would have using 100 steps per minute. Even with this individualized algorithm, Fitbit captured fewer minutes of MVPA than AGH. Average stride length in another knee OA sample was 1.05 meters,[46] corresponding to a 79 steps per minute MVPA threshold, a cadence well below the established 100 steps per minute guideline. This highlights the importance of using characteristic gait parameters of the sample if it is infeasible to tailor thresholds to individual metrics.

Our study was limited by its small sample size (15 participants). However, the study unit was days, yielding 160 valid Fitbit person-days. We were unable to examine limited mobility within this population, as only one participant used a walker. The accuracy of accelerometers might be susceptible to users’ mobility limitations, particularly in the OA population. Future studies should validate accelerometers in individuals utilizing ambulatory assistance.

Our ability to validate MVPA was limited by the low PA levels of this sample, likely representative of the knee OA population more broadly, in free-living conditions. In the AGH versus Fitbit comparison, both devices captured zero minutes of MVPA in 50% of the valid person-days. The low MVPA levels may be due to physical functional limitations. For persons with knee OA, knee pain is associated with less time spent in MVPA but not less time spent in light activity[47]. In addition, we did not validate the devices at the minute-level, due to potential alignment problems, but instead examined the summarized day or week data.

Fitbit’s HR detection feature allowed us to identify minutes when the participant wore the device but took zero steps. Incorporating HR data captured more wear time relative to the step-based algorithm that categorized wear periods inappropriately as sedentary time. Without the HR feature, it is challenging to discern whether zero recorded steps represent non-wear or sedentary behavior on the Fitbit. ActiGraphs are sensitive to subtle motions and postural shifts, whereas the Fitbit, which records steps, does not detect and capture these motions. However, we were not able to correct for potential artifacts due to variation in positioning on wrists for either device. The previously-established step-based algorithm captured 14% less wear time than the HR-based algorithm. HR captured by commercial accelerometers can improve accuracy in discerning non-wear from sedentary behaviors and is closely related to ActiGraph measures. Future studies using accelerometers that capture HR may incorporate these data when deriving non-wear time. Using HR offers a more complete understanding of the distribution of time spent in sedentary behaviors, which may be useful in interventions to increase light activity.

The HR detection feature, ability to download subject data remotely, and relatively affordable pricing make the Fitbit Charge 2 a scalable means to measure objective PA in large-scale interventions. This commercially-available accelerometer is appropriate for measuring changes in PA-related metrics due to interventions, but, if the Fitbit is used to measure PA itself in subjects with knee OA, appropriate adjustments for each measure should be applied.

## Supporting information

S1 File Fig A. Pairwise comparisons of hours in light activity per day. The black line represents the regression line, and the grey line represents a 45-degree line. Comparisons for Fitbit versus ActiGraph hip (AGH), ActiGraph wrist (AGW) versus AGH, and Fitbit versus AGW are presented in panels a, b, and c, respectively. Fig B. Daily hours of wear time measured by the step-based and heart rate (HR) Fitbit algorithms. Fitbit processing algorithm (step-based versus HR) comparison for daily wear time (hours) in each pairwise comparison. The relationship between wear time as measured by the step- and heart rate-based Fitbit wear time algorithms is HR wear hours = 0.9753*step-based wear hours + 1.84. Fig C. Enrollment procedure schematic. The flow diagram outlines the procedure scheme following when recruiting and enrolling participants in the study. Table A. Regression equations for each pairwise comparison with Fitbit, wrist ActiGraph (AGW), and hip ActiGraph (AGH). The comparisons are presented as follows: sedentary time: Fig 2; MVPA: Fig 3; steps per day: Fig 4; light activity: Fig A. Table B. Comparison of average day-level physical activity measured by the Fitbit and hip ActiGraph (AGH) stratified by <7,500 and ≥7,500 steps per day as measured by AGH. Table C. Comparison of average day-level physical activity measured by the wrist ActiGraph (AGW) and hip ActiGraph (AGH) stratified by <7,500 and ≥7,500 steps per day as measured by AGH. Table D. Schedule for trial participants. *Questionnaire is comprised of the “Baseline Health Questionnaire” and KOOS; Assessment is comprised of the 30-step walk test (completed two times). Table E. Wear and compensation schedule for trial participants.
(DOCX)

S2 File Contains Baseline questionnaire. (PDF)

S3 File Contains KOOS instrument. (PDF)

S4 File Contains post-study survey. (PDF)

S5 File Data repository; minimal anonymized dataset necessary to replicate study findings. (XLSX)
