# Supplementary material for: Validation of the Fitbit Charge 2 compared to the ActiGraph GT3X+ in older adults with knee osteoarthritis in free-living conditions
Source: PLoS One. 2019 Jan 30;14(1):e0211231. doi: 10.1371/journal.pone.0211231 (PMC6353569; doi:10.1371/journal.pone.0211231)
Supplement: S1 File — Fig A. Pairwise comparisons of hours in light activity per day. The black line represents the regression line, and the grey line represents a 45-degree line. Comparisons for Fitbit versus ActiGraph hip (AGH), ActiGraph wrist (AGW) versus AGH, and Fitbit versus AGW are presented in panels a, b, and c, respectively. Fig B. Daily hours of wear time measured by the step-based and heart rate (HR) Fitbit algorithms. Fitbit processing algorithm (step-based versus HR) comparison for daily wear time (hours) in each pairwise comparison. The relationship between wear time as measured by the step- and heart rate-based Fitbit wear time algorithms is HR wear hours = 0.9753*step-based wear hours + 1.84. Fig C. Enrollment procedure schematic. The flow diagram outlines the procedure scheme following when recruiting and enrolling participants in the study. Table A. Regression equations for each pairwise comparison with Fitbit, wrist ActiGraph (AGW), and hip ActiGraph (AGH). The comparisons are presented as follows: sedentary time: Fig 2; MVPA: Fig 3; steps per day: Fig 4; light activity: Fig A. Table B. Comparison of average day-level physical activity measured by the Fitbit and hip ActiGraph (AGH) stratified by <7,500 and ≥7,500 steps per day as measured by AGH. Table C. Comparison of average day-level physical activity measured by the wrist ActiGraph (AGW) and hip ActiGraph (AGH) stratified by <7,500 and ≥7,500 steps per day as measured by AGH. Table D. Schedule for trial participants. *Questionnaire is comprised of the “Baseline Health Questionnaire” and KOOS; Assessment is comprised of the 30-step walk test (completed two times). Table E. Wear and compensation schedule for trial participants. (DOCX) [file pone.0211231.s001.docx]

S1 Supplementary Materials

S1 Figure A: Pairwise comparisons of hours in light activity per day.

S1 Figure B: Daily hours of wear time measured by the step-based and heart rate (HR) Fitbit algorithms.

S1 Figure C: Enrollment procedure schematic.

S1 Table A: Regression equations for each pairwise comparison with Fitbit, wrist ActiGraph (AGW), and hip ActiGraph (AGH).

S1 Table B: Comparison of average day-level physical activity measured by the Fitbit and hip ActiGraph (AGH) stratified by <7,500 and ≥7,500 steps per day as measured by AGH.

S1 Table C: Comparison of average day-level physical activity measured by the wrist ActiGraph (AGW) and hip ActiGraph (AGH) stratified by <7,500 and ≥7,500 steps per day as measured by AGH.

S1 Table D: Schedule for trial participants.

S1 Table E: Wear and compensation schedule for trial participants.

S1 Figure A.


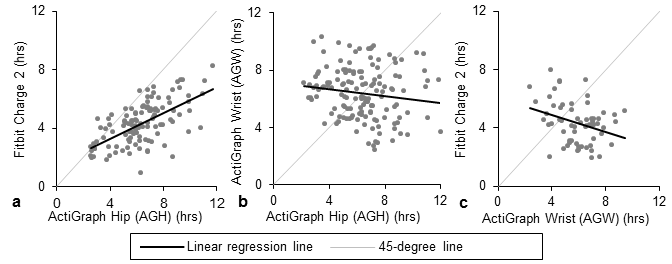


S1 Figure B.


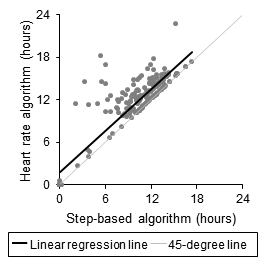


S1 Figure C.





S1 Table A.

| Measure | Fitbit versus AGH | | AGW versus AGH | | Fitbit versus AGW | |
| --- | --- | --- | --- | --- | --- | --- |
|  | *Equation* | *R^2^* | *Equation* | *R^2^* | *Equation* | *R^2^* |
| Sedentary time (hours) | Fitbit= 0.794*AGH+3.4 | 0.4308 | AGW= 0.296*AGH+0.2 | 0.1850 | Fitbit= 1.303*AGW+5.9 | 0.3584 |
| Light activity (hours) | Fitbit= 0.437*AGH+1.5 | 0.3829 | AGW= -0.127*AGH+7.2 | 0.0217 | Fitbit= -0.329*AGW+7.4 | 0.0945 |
| MVPA (minutes) | Fitbit= 0.330*AGH+5 | 0.1284 | AGW= 1.927*AGH+264 | 0.1440 | Fitbit= 0.025*AGW+5 | 0.0210 |
| Steps per day | Fitbit= 1.258*AGH+336 | 0.6618 | AGW= 0.902*AGH+4,529 | 0.6382 | Fitbit= 0.984*AGW–2,212 | 0.4781 |

S1 Table B.

|  | <7,500 steps per day | | ≥7,500 steps per day | |
| --- | --- | --- | --- | --- |
| N (person-days) | 88 | | 26 | |
|  | **Fitbit** | **AGH** | **Fitbit** | **AGH** |
| Sedentary time (minutes) |  |  |  |  |
| Mean | 531 | 413 | 445 | 300 |
| SD | 102 | 94 | 181 | 103 |
| Median | 512 | 426 | 422 | 291 |
| Difference vs. AGH | 118 | - | 145 | - |
| % bias vs. AGH | 34 | - | 49 | - |
| MVPA, bouted (minutes) |  |  |  |  |
| Mean | 6 | 7 | 24 | 38 |
| SD | 15 | 14 | 29 | 29 |
| Median | 0 | 0 | 12 | 30 |
| Difference vs. AGH | -2 | - | -14 | - |
| Steps |  |  |  |  |
| Mean | 5,273 | 3,944 | 11,670 | 8,942 |
| SD | 2,783 | 1,784 | 4,268 | 1,223 |
| Median | 5,063 | 3,734 | 11,848 | 8,427 |
| Difference vs. AGH | 1,329 | - | 2,728 | - |
| % bias vs. AGH | 42 | - | 29 | - |
| Person-weeks (n [%]) achieving MVPA thresholds | 26 | | 12 | |
| ≥150 min/week | 0 (0) | 0 (0) | 1 (8) | 5 (42) |
| ≥45 min/week | 4 (15) | 10 (38) | 8 (67) | 8 (67) |

S1 Table C.

|  | <7,500 steps per day | | ≥7,500 steps per day | |
| --- | --- | --- | --- | --- |
| N (person-days) | 110 | | 33 | |
|  | **AGW** | **AGH** | **AGW** | **AGH** |
| Sedentary time (minutes) |  |  |  |  |
| Mean | 140 | 448 | 132 | 311 |
| SD | 68 | 103 | 113 | 98 |
| Median | 132 | 454 | 77 | 299 |
| Difference vs. AGH | -308 | - | -180 | - |
| % bias vs. AGH | -69 | - | -59 | - |
| MVPA, bouted (minutes) |  |  |  |  |
| Mean | 268 | 9 | 400 | 46 |
| SD | 123 | 18 | 155 | 37 |
| Median | 248 | 0 | 413 | 35 |
| Difference vs. AGH | 259 | - | 354 | - |
| Steps |  |  |  |  |
| Mean | 7,983 | 3,783 | 12,958 | 9,500 |
| SD | 2,747 | 1,823 | 2,111 | 1,340 |
| Median | 7,743 | 3,398 | 12,762 | 9,644 |
| Difference vs. AGH | 4,200 | - | 3,458 | - |
| % bias vs. AGH | 139 | - | 38 | - |
| Person-weeks (n [%]) achieving MVPA thresholds | 26 | | 13 | |
| ≥150 min/week | 26 (100) | 1 (4) | 13 (100) | 5 (38) |
| ≥45 min/week | 26 (100) | 11 (42) | 13 (100) | 8 (62) |

S1 Table D.

|  | Informed Consent | Baseline Questionnaire + Assessment* | Daily Reminder to Wear + Charge | Reminder to Return Devices | Post-Study Questionnaire |
| --- | --- | --- | --- | --- | --- |
| Baseline Visit | X | X |  |  |  |
| Study Duration (Weeks 1-4) |  |  | X |  |  |
| Post-Study |  |  |  | X | X |

S1 Table E.

|  | Baseline | Week 1 | Week 2 | Week 3 | Week 4 | Completion |
| --- | --- | --- | --- | --- | --- | --- |
| ActiGraph Waist |  | X | X | X | X |  |
| ActiGraph Wrist |  |  | X | X |  |  |
| Fitbit Wrist |  |  |  | X | X |  |
| **Incentive** | $25 | $3/Day | $5/Day | $7/day | $5/Day | $30 |
| **Maximum Value** | $25 | $21 | $35 | $49 | $35 | $30 |
