# Supplementary material for: Validation of the Fitbit Charge 2 compared to the ActiGraph GT3X+ in older adults with knee osteoarthritis in free-living conditions
Source: PLoS One. 2019 Jan 30;14(1):e0211231. doi: 10.1371/journal.pone.0211231 (PMC6353569; doi:10.1371/journal.pone.0211231)
Supplement: S2 File — (PDF) [file pone.0211231.s002.pdf]

Baseline Participant Questionnaire Subject Initials: ______

Date Completed: ___ ___/___ ___/___ ___(*MM/DD/YY*)

To be completed by the **study participant**.

This questionnaire asks you about your medication and medical condition history. Please answer every question. Fill in only one answer per question unless instructed otherwise.

A. MEDICATION INFORMATION

1. In the **past week**, did you take any medication(s) for your knee pain?

0☐ No 1☐ Yes

1a. If "Yes", are you taking…

I. Acetaminophen (Tylenol, Panadol)?

0☐ No 1☐ Yes

II. Non-steroidal anti-inflammatory drugs (NSAIDs)?

0☐ No 1☐ Yes →

1. *Please check the one(s) you took*:
   - 1☐ Aspirin (Bayer, Excedrin, Bufferin)
   - 2☐ Ibuprofen (Advil, Motrin, Nuprin)
   - 3☐ Naproxen (Aleve, Naprosyn, Vimovo)
   - 4☐ Ketoprofen (Actron, Orudis)
   - 5☐ Diclofenac (Voltaren)
   - 6☐ Celecoxib (Celebrex)
   - 7☐ Nabumetone (Relafen)
   - 8☐ Oxaprozin (Daypro, Dayrun, Duraprox)
   - 9☐ Etodolac (Lodine, Lodine XL)
   - 10☐ Meloxicam (Mobic)
   - 11☐ Other. Please specify:

   ____

III. Opioids?

0☐ No 1☐ Yes →

1. *Please check the one(s) you took:*
   - 0☐ Oxycodone (OxyContin, Oxecta, Roxicodone)
   - 1☐ Oxycodone/Acetaminophen (Percocet, Endocet, Roxicet)
   - 2☐ Tramadol
   - 3☐ Hydrocodone (Hysingla ER, Zohydro ER)
   - 4☐ Hydrocodone/Acetaminophen (Lorcet, Lortab, Norco, Vicodin)
   - 5☐ Codeine
   - 6☐ Hydromorphone (Dilaudid, Exalgo)
   - 7☐ Methadone (Dolophine, Methadose)
   - 8☐ Meperidine (Demerol)
   - 9☐ Fentanyl (Antiq, Duragesic, Fentora)
   - 10☐ Other. Please specify:

   ____

Please flip to the back →

2. Other than vitamins or supplements, are you currently taking other medications?

0☐ No 1☐ Yes

2a. If "Yes", please list the medications you took in the **past week**:

_____________________ _____________________

_____________________ _____________________

_____________________ _____________________

_____________________ _____________________

**B. MEDICAL HISTORY**	**No**	**Yes**
**3a.** Have you ever been told by a medical professional that you have high blood pressure?	0☐	1☐
**I.** In the **past week**, have you taken medication to control your blood pressure?<br>If "Yes", please specify the medication(s):<br>_____________________<br>_____________________	0☐	1☐
**3b.** Have you ever been told by a medical professional that you had *congestive heart failure*?	0☐	1☐
**3c.** Have you ever been told by a medical professional that you had a *heart attack*?	0☐	1☐
**3d.** Have you ever undergone coronary artery bypass surgery (CABG)?	0☐	1☐
**3e.** Have you ever had a procedure to unblock vessels in your heart, such as coronary stenting or an angioplasty?	0☐	1☐
**3f.** Have you ever been told by a medical professional that you had a *stroke*?	0☐	1☐
**3g.** Have you ever been told by a medical professional that you have *diabetes*?	0☐	1☐
**I.** In the **past week**, have you taken *insulin injections* to control your diabetes?	0☐	1☐
**II.** In the **past week**, have you taken *pills or other medications* to control your diabetes?<br>If "Yes" to a *diabetes treatment*, please specify the medication name(s) below:<br>_____________________<br>_____________________	0☐	1☐

Date Completed: ___ ___/___ ___/___ ___*(MM/DD/YY)*

To be completed by the **research assistant**.

**C. PARTICIPANT INFORMATION**
**1.** Age:<br>a. Month of Birth (MM): ____________________<br>b. Year of Birth (YYYY): ____________________
**2.** Sex:<br>0☐ Female<br>1☐ Male
**3.** Height:<br>a. _______________ft.<br>b. _______________in.
**4.** Weight: __________________lbs.
**5.** 30-Step Walk Length Results (in inches):<br>*Use half-inch increments.*<br>a. 1st Walk: ______________ in. 2nd Walk: ______________ in.
