# Supplementary material for: Validation of the Fitbit Charge 2 compared to the ActiGraph GT3X+ in older adults with knee osteoarthritis in free-living conditions
Source: PLoS One. 2019 Jan 30;14(1):e0211231. doi: 10.1371/journal.pone.0211231 (PMC6353569; doi:10.1371/journal.pone.0211231)
Supplement: S3 File — (PDF) [file pone.0211231.s003.pdf]

KOOS KNEE SURVEY

Today's date: _____/______/______ Date of birth: _____/______/______

Name: ____

INSTRUCTIONS: This survey asks for your view about your knee. This information will help us keep track of how you feel about your knee and how well you are able to perform your usual activities.
Answer every question by ticking the appropriate box, only one box for each question. If you are unsure about how to answer a question, please give the best answer you can.

Symptoms

These questions should be answered thinking of your knee symptoms during the **last week**.

S1. Do you have swelling in your knee?

Never	Rarely	Sometimes	Often	Always
☐	☐	☐	☐	☐

S2. Do you feel grinding, hear clicking or any other type of noise when your knee moves?

Never	Rarely	Sometimes	Often	Always
☐	☐	☐	☐	☐

S3. Does your knee catch or hang up when moving?

Never	Rarely	Sometimes	Often	Always
☐	☐	☐	☐	☐

S4. Can you straighten your knee fully?

Always	Often	Sometimes	Rarely	Never
☐	☐	☐	☐	☐

S5. Can you bend your knee fully?

Always	Often	Sometimes	Rarely	Never
☐	☐	☐	☐	☐

Stiffness

The following questions concern the amount of joint stiffness you have experienced during the **last week** in your knee. Stiffness is a sensation of restriction or slowness in the ease with which you move your knee joint.

S6. How severe is your knee joint stiffness after first wakening in the morning?

None	Mild	Moderate	Severe	Extreme
☐	☐	☐	☐	☐

S7. How severe is your knee stiffness after sitting, lying or resting **later in the day**?

None	Mild	Moderate	Severe	Extreme
☐	☐	☐	☐	☐

Pain

P1. How often do you experience knee pain?

Never	Monthly	Weekly	Daily	Always
☐	☐	☐	☐	☐

What amount of knee pain have you experienced the **last week** during the following activities?

P2. Twisting/pivoting on your knee

None	Mild	Moderate	Severe	Extreme
☐	☐	☐	☐	☐

P3. Straightening knee fully

None	Mild	Moderate	Severe	Extreme
☐	☐	☐	☐	☐

P4. Bending knee fully

None	Mild	Moderate	Severe	Extreme
☐	☐	☐	☐	☐

P5. Walking on flat surface

None	Mild	Moderate	Severe	Extreme
☐	☐	☐	☐	☐

P6. Going up or down stairs

None	Mild	Moderate	Severe	Extreme
☐	☐	☐	☐	☐

P7. At night while in bed

None	Mild	Moderate	Severe	Extreme
☐	☐	☐	☐	☐

P8. Sitting or lying

None	Mild	Moderate	Severe	Extreme
☐	☐	☐	☐	☐

P9. Standing upright

None	Mild	Moderate	Severe	Extreme
☐	☐	☐	☐	☐

Function, daily living

The following questions concern your physical function. By this we mean your ability to move around and to look after yourself. For each of the following activities please indicate the degree of difficulty you have experienced in the **last week** due to your knee.

A1. Descending stairs

None	Mild	Moderate	Severe	Extreme
☐	☐	☐	☐	☐

A2. Ascending stairs

None	Mild	Moderate	Severe	Extreme
☐	☐	☐	☐	☐

For each of the following activities please indicate the degree of difficulty you have experienced in the **last week** due to your knee.

A3. Rising from sitting

None	Mild	Moderate	Severe	Extreme
☐	☐	☐	☐	☐

A4. Standing

None	Mild	Moderate	Severe	Extreme
☐	☐	☐	☐	☐

A5. Bending to floor/pick up an object

None	Mild	Moderate	Severe	Extreme
☐	☐	☐	☐	☐

A6. Walking on flat surface

None	Mild	Moderate	Severe	Extreme
☐	☐	☐	☐	☐

A7. Getting in/out of car

None	Mild	Moderate	Severe	Extreme
☐	☐	☐	☐	☐

A8. Going shopping

None	Mild	Moderate	Severe	Extreme
☐	☐	☐	☐	☐

A9. Putting on socks/stockings

None	Mild	Moderate	Severe	Extreme
☐	☐	☐	☐	☐

A10. Rising from bed

None	Mild	Moderate	Severe	Extreme
☐	☐	☐	☐	☐

A11. Taking off socks/stockings

None	Mild	Moderate	Severe	Extreme
☐	☐	☐	☐	☐

A12. Lying in bed (turning over, maintaining knee position)

None	Mild	Moderate	Severe	Extreme
☐	☐	☐	☐	☐

A13. Getting in/out of bath

None	Mild	Moderate	Severe	Extreme
☐	☐	☐	☐	☐

A14. Sitting

None	Mild	Moderate	Severe	Extreme
☐	☐	☐	☐	☐

A15. Getting on/off toilet

None	Mild	Moderate	Severe	Extreme
☐	☐	☐	☐	☐

For each of the following activities please indicate the degree of difficulty you have experienced in the **last week** due to your knee.

A16. Heavy domestic duties (moving heavy boxes, scrubbing floors, etc)

None	Mild	Moderate	Severe	Extreme
☐	☐	☐	☐	☐

A17. Light domestic duties (cooking, dusting, etc)

None	Mild	Moderate	Severe	Extreme
☐	☐	☐	☐	☐

Function, sports and recreational activities

The following questions concern your physical function when being active on a higher level. The questions should be answered thinking of what degree of difficulty you have experienced during the **last week** due to your knee.

SP1. Squatting

None	Mild	Moderate	Severe	Extreme
☐	☐	☐	☐	☐

SP2. Running

None	Mild	Moderate	Severe	Extreme
☐	☐	☐	☐	☐

SP3. Jumping

None	Mild	Moderate	Severe	Extreme
☐	☐	☐	☐	☐

SP4. Twisting/pivoting on your injured knee

None	Mild	Moderate	Severe	Extreme
☐	☐	☐	☐	☐

SP5. Kneeling

None	Mild	Moderate	Severe	Extreme
☐	☐	☐	☐	☐

Quality of Life

Q1. How often are you aware of your knee problem?

Never	Monthly	Weekly	Daily	Constantly
☐	☐	☐	☐	☐

Q2. Have you modified your life style to avoid potentially damaging activities to your knee?

Not at all	Mildly	Moderately	Severely	Totally
☐	☐	☐	☐	☐

Q3. How much are you troubled with lack of confidence in your knee?

Not at all	Mildly	Moderately	Severely	Extremely
☐	☐	☐	☐	☐

Q4. In general, how much difficulty do you have with your knee?

None	Mild	Moderate	Severe	Extreme
☐	☐	☐	☐	☐

Thank you very much for completing all the questions in this questionnaire.
