# Supplementary material for: Validation of the Fitbit Charge 2 compared to the ActiGraph GT3X+ in older adults with knee osteoarthritis in free-living conditions
Source: PLoS One. 2019 Jan 30;14(1):e0211231. doi: 10.1371/journal.pone.0211231 (PMC6353569; doi:10.1371/journal.pone.0211231)
Supplement: S4 File — (PDF) [file pone.0211231.s004.pdf]

Post-Study Satisfaction Survey
ActiGraph and Fitbit Wear
January 10, 2017

Today's date : _____ / _____ / __________ *(MM/DD/YYYY)*

A. VISIT INFORMATION

1. Did you find the **Fitbit** easy to wear on your **wrist**?
   - 0☐ No
   - 1☐ Yes

   If *no*, what made the device difficult to wear?

2. Were the instructions for how to wear the **Fitbit** on your **wrist** clear?
   - 0☐ No
   - 1☐ Yes

   If *no*, what can we do to make the instructions more clear?

3. Did you experience any discomfort wearing the **Fitbit**?
   - 0☐ No
   - 1☐ Yes

   If *yes*, please describe:

4. Did you find the **ActiGraph** easy to wear on your **wrist**?
   - 0☐ No
   - 1☐ Yes

   If *no*, what made the device difficult to wear?

5. Were the instructions for how to wear the **ActiGraph** on your **wrist** clear?
   - 0☐ No
   - 1☐ Yes

   If *no*, what can we do to make the instructions more clear?

6. Did you experience any discomfort while wearing the **Actigraph** on your **wrist**?
   - 0☐ No
   - 1☐ Yes

   If *yes*, please describe:

7. Did you find the **ActiGraph** easy to wear on your **waist**?
   - 0☐ No
   - 1☐ Yes

   If *no*, what made the device difficult to wear?

8. Were the instructions for how to wear the **ActiGraph** on your **waist** clear?
   - 0☐ No
   - 1☐ Yes

   If *no*, what can we do to make the instructions more clear?

9. Did you experience any discomfort while wearing the **Actigraph** on your **waist**?
   - 0☐ No
   - 1☐ Yes

   If *yes*, please describe:

10. Were you satisfied with communication with the study staff?
    - 0☐ No
    - 1☐ Yes

11. Overall, were you satisfied with your participation in the study?
    - 0☐ No
    - 1☐ Yes

12. Is there anything study staff could have done to improve your experience?
